# Supplementary material for: A comparison of species composition and community assemblage of secondary forests between the birch and pine-oak belts in the mid-altitude zone of the Qinling Mountains, China
Source: PeerJ. 2016 Apr 21;4:e1900. doi: 10.7717/peerj.1900 (PMC4846807; doi:10.7717/peerj.1900)
Supplement: Supplemental Information 3 [file peerj-04-1900-s003.pdf]

The introduction of species abundance distribution

Chai Zongzheng

Email: chaizz@126.com

College of Forestry, Northwest A & F University, Yangling 712100, China.

The following six Species abundance distribution (SAD) models were considered: broken-stick, niche-preemption, log-normal, Zipf, Zipf-Mandelbrot, and neutral-theory models (Table 1). Further details and comments of other SAD models are described by McGill et al. (2007) and Wilson (1991).

Table 1. Six main species abundance distribution models.

Model	Equation		Reference
Broken-stick	$\hat{a}_r = \frac{N}{S}\sum_{k=r}^{S}\frac{1}{k}$	(1)	MacArthur (1957)
Niche-preemption	$\hat{a}_r = N\alpha(1-a)^{r-1}$	(2)	Motomura (1932)
Log-normal	$\hat{a}_r = \exp[\log(u) + \log(\sigma)\Phi]$	(3)	Preston (1948)
Zipf	$\hat{a}_r = N\hat{p}_1 r^{\gamma}$	(4)	Frontier (1987)
Zipf-Mandelbrot	$\hat{a}_r = Nc(r+\beta)^{\gamma}$	(5)	
Neutral-theory	$\phi_n = \theta\frac{J!}{n!\,(J-n)!}\frac{\Gamma(\gamma)}{\Gamma(J+\gamma)}\int_0^{\gamma}\frac{\Gamma(n+y)}{\Gamma(1+y)}\frac{\Gamma(J-n+\gamma-y)}{\Gamma(\gamma-y)}\exp(-y\theta/\gamma)dy$	(6)	Hubbell (2001)

Note: $\hat{a}_r$ is the expected abundance of species at rank r, S is the number of species, N is the number of individuals, Φ is a standard normal function, $\hat{p}_1$ is the estimated proportion of the most abundant species, and $\alpha, \sigma, \gamma, \beta$ and c are the estimated parameters in each model. In neutral-theory model, where $\Gamma(z) = \int_0^{\infty} t^{z-1} e^{-t}dt$ which is equal to (z-1)!, for integer z and $\gamma = \frac{m(J-1)}{1-m}$, θ is fundamental diversity number, m is migration rate.

Broken-stick model: This model was first proposed by MacArthur (1957). Its analogy of placing *s*-points randomly on a line of unit length and simultaneously breaking it at those points into *s* lengths can be rephrased as a group of *s* series. The lengths of the segments represent the “niche sizes” of the species. According to the model, the expected size of the *r*th species, and $\hat{a}_r$, the expected abundance of species of species at rank *r*, are shown in equation (1) in Table 1. The mathematical proof of this model can be found in Pielou (1975).

Niche-preemption model This model was proposed by (Motomura, 1932) and assumes that the percentage of the total niche occupied by the first species is α, the second species occupied a percentage α of the reminder, $\alpha(1-\alpha)$, and so on. The expected abundance for the *r*th species is equation (2) in Table 1.

Log-normal model A log-normal distribution is defined as a distribution whose variate conforms to the normal laws of probability. For SADs, the log-normal distribution characterizes a sample with relatively low abundance or very rare species (Matthews and Whittaker, 2014). Preston (1948) introduced the log-normal SAD by demonstrating a good fit to a large number of data sets covering a number of different communities. See equation (3) in Table 1.

Zipf and Zipf-Mandelbrot model The Mandelbrot model was originally developed for information systems, assessing the cost of information (Frontier, 1985). In plant communities, the presence of a species can be seen as dependent on previous physical conditions and previous species presences – these are the costs. Pioneer species have a low cost, requiring few prior conditions. Late successional species have a high cost, viz. the energy, time, and organization of the ecosystem required before they can invade. On this basis they will be rare (Frontier, 1987). These differences between species give a Zipf or Zipf-Mandelbrot distribution, equations (4) and (5) in Table 1, respectively. The assumption is that a species is very likely to invade once its necessary conditions are met (Wilson, 1991).

Neutral-theory model Hubbell (2001) noted that the relative abundance of species within – and the species diversity of – a community can be explained through neutral drift of individual species abundances. The model contends that the number of individuals in a metacommunity is constant, that is, all available resources in the community are saturated. This is the zero-sum

assumption: if an individual dies and a portion of the resource becomes available, it will be immediately taken up by a new individual, and the community size remains constant. See equation (6) in Table 1.

Reference:

Frontier S (1985). Diversity and structure in aquatic ecosystems.Oceanogr.Mar.Biol.Ann.Rev.23:253-312.

Frontier S (1987). Applications of fractal theory to ecology. In:Legender, P.(ed) Developments in Numerical Ecology, pp.357-378. Springer-Verlag, Berlin.

Hubbell SP (2001). The unified neutral theory of biodiversity and biogeography. *Princeton Univ. Press, Princeton.*

MacArthur RH (1957). On the relative abundance of bird species. Proceedings of the National Academy of Sciences, USA, 43: 293–295.

Matthews TJ, Whittaker RJ (2014). Neutral theory and the species abundance distribution: Recent developments and prospects for unifying niche and neutral perspectives. Ecol Evol 4:2263-2277.

McGill BJ, Etienne RS, Gray JS, Alonso D, Anderson MJ, Benecha HK, Dornelas M, Enquist BJ, Green JL, He FL, Hurlbert AH, Magurran AE, Marquet PA, Maurer BA, Ostling A, Soykan CU, Ugland KI, White EP (2007).

Motomura I (1932). On the statistical treatment of communities. Zool.Mag., 44:379-383

Pielou EC (1975). Ecological Diversity. Wiley, New York.

Preston F (1948). The commonness and rarity of species. Ecology 29:254-283.

Wilson JB (1991). Methods for fitting dominance/diversity curves. J Veg Sci 2:35-46.
