# Supplementary material for: A comparison of species composition and community assemblage of secondary forests between the birch and pine-oak belts in the mid-altitude zone of the Qinling Mountains, China
Source: PeerJ. 2016 Apr 21;4:e1900. doi: 10.7717/peerj.1900 (PMC4846807; doi:10.7717/peerj.1900)
Supplement: Supplemental Information 4 [file peerj-04-1900-s004.docx]

**Table S1** Species composition and importance value (IV) index of the typical secondary forests in mid-mountain zones in Qinling Mountains, China.

| Chinese name | Scientific name | Family | Abundance | Importance value (%) | | | | | | | | | | | |
| --- | --- | --- | --- | --- | --- | --- | --- | --- | --- | --- | --- | --- | --- | --- | --- |
|  |  |  |  | Forest stand | | | | | | | | | | Forest belt | |
|  |  |  |  | BA | TCBA | PABA | CTBA | AFBA | PA | PT | QA | PAQA | PTQA | Birch belt | Pine-oak belt |
| 锐齿栎 | *Quercus aliena* var. *acutiserrata* Maxim. | Fagaceae | 1021 | 2.29 | 3.75 | 5.27 | 7.77 | 10.17 | 8.16 | 12.87 | 59.49 | 23.32 | 26.92 | 5.85 | 26.15 |
| 油松 | *Pinus tabulaeformis* Carr. | Pinaceae | 719 | 0.42 | 0.00 | 0.42 | 0.00 | 0.00 | 1.66 | 45.25 | 13.29 | 15.54 | 36.74 | 0.17 | 22.50 |
| 华山松 | *Pinus armandii* Franch. | Pinaceae | 707 | 11.78 | 14.84 | 13.00 | 6.25 | 5.06 | 38.94 | 8.34 | 6.26 | 34.12 | 12.59 | 10.19 | 20.05 |
| 漆树 | *Toxicodendron vernicifluum* (Stokes) F. A. Barkl. | Anacardiaceae | 408 | 3.47 | 4.56 | 9.54 | 8.77 | 14.93 | 17.93 | 10.38 | 4.36 | 7.75 | 10.93 | 8.25 | 10.27 |
| 青榨槭 | *Acer davidii* Franch. | Aceraceae | 362 | 6.49 | 8.12 | 10.92 | 8.91 | 9.38 | 0.83 | 0.92 | 0.00 | 2.18 | 0.00 | 8.76 | 0.79 |
| 铁杉 | *Tsuga chinensis* (Franch.) Pritz. | Pinaceae | 253 | 7.48 | 9.50 | 9.61 | 3.62 | 4.22 | 6.12 | 4.62 | 0.00 | 0.00 | 0.00 | 6.89 | 2.15 |
| 红桦 | *Betula albo-sinensis* Burk. | Betulaceae | 239 | 11.75 | 7.68 | 6.47 | 15.99 | 11.23 | 4.47 | 1.32 | 0.00 | 2.45 | 0.00 | 10.63 | 1.65 |
| 花楸 | *Sorbus pohuashanensis* (Hance) Hedl. | Rosaceae | 229 | 6.79 | 7.33 | 7.96 | 5.47 | 7.67 | 4.01 | 0.00 | 0.00 | 0.00 | 0.00 | 7.04 | 0.80 |
| 鹅耳枥 | *Carpinus turczaninowii* Hance | Betulaceae | 166 | 4.05 | 3.24 | 1.47 | 10.10 | 5.41 | 2.05 | 4.89 | 4.52 | 1.44 | 4.52 | 4.85 | 3.48 |
| 茶条槭 | *Acer ginnala* Maxim. | Aceraceae | 132 | 6.89 | 3.84 | 0.89 | 2.44 | 1.13 | 0.00 | 1.01 | 0.00 | 0.00 | 0.00 | 3.04 | 0.20 |
| 小叶杨 | *Populus simonii* Carr. | Salicaceae | 122 | 0.00 | 2.15 | 8.72 | 0.00 | 0.00 | 0.45 | 0.00 | 0.00 | 0.00 | 0.44 | 2.17 | 0.18 |
| 栓皮栎 | *Quercus variabilis* Blume | Fagaceae | 116 | 2.66 | 4.85 | 2.99 | 4.47 | 4.75 | 0.72 | 0.00 | 0.00 | 0.00 | 0.00 | 3.94 | 0.14 |
| 托叶樱桃 | *Cerasus stipulacea* (Maxim.) Yü et Li | Rosaceae | 106 | 2.98 | 3.63 | 2.78 | 7.47 | 0.00 | 0.00 | 0.00 | 0.00 | 0.00 | 0.00 | 3.37 | 0.00 |
| 五裂槭 | *Acer oliverianum* Pax | Aceraceae | 77 | 2.36 | 3.64 | 2.59 | 1.78 | 3.76 | 1.22 | 0.00 | 0.00 | 0.94 | 0.00 | 2.83 | 0.43 |
| 毛花槭 | *Acer erianthum* Schwer. | Aceraceae | 70 | 4.91 | 0.00 | 1.00 | 0.36 | 0.00 | 0.00 | 0.00 | 0.00 | 0.00 | 0.00 | 1.25 | 0.00 |
| 刺楸 | *Kalopanax septemlobus* (Thunb.) Koidz. | Araliaceae | 70 | 0.00 | 5.07 | 0.31 | 3.97 | 1.66 | 0.43 | 0.00 | 1.06 | 0.00 | 0.00 | 2.20 | 0.30 |
| 巴山冷杉 | *Abies fargesii* Franch. | Pinaceae | 67 | 0.64 | 1.88 | 0.64 | 1.34 | 5.35 | 0.00 | 0.00 | 0.00 | 0.90 | 0.00 | 1.97 | 0.18 |
| 榛子 | *Corylus heterophylla* Fisch. ex Trautv. | Betulaceae | 62 | 1.43 | 2.17 | 2.47 | 0.79 | 3.49 | 0.71 | 1.28 | 0.00 | 0.70 | 0.96 | 2.07 | 0.73 |
| 五角枫 | *Acer elegantulum* Fang et P. L. Chiu | Aceraceae | 61 | 3.55 | 0.41 | 1.54 | 0.36 | 1.09 | 0.00 | 0.00 | 0.00 | 0.00 | 0.00 | 1.39 | 0.00 |
| 光皮桦 | *Betula luminifera* H. Winkl. | Betulaceae | 61 | 6.55 | 2.68 | 1.03 | 0.37 | 3.11 | 0.00 | 0.00 | 0.00 | 0.00 | 0.00 | 2.75 | 0.00 |
| 三桠乌药 | *Lindera obtusiloba* Bl. | Lauraceae | 58 | 0.66 | 0.00 | 0.00 | 0.00 | 0.84 | 2.68 | 1.00 | 3.25 | 2.54 | 0.00 | 0.30 | 1.89 |
| 木姜子 | *Litsea pungens* Hemsl. | Lauraceae | 57 | 1.08 | 2.56 | 0.99 | 0.00 | 2.43 | 0.00 | 1.72 | 0.00 | 0.89 | 0.00 | 1.41 | 0.52 |
| 楸树 | *Catalpa bungei* C. A. Mey | Bignoniaceae | 51 | 1.34 | 0.00 | 3.71 | 2.62 | 0.00 | 0.71 | 0.00 | 0.00 | 0.00 | 0.00 | 1.53 | 0.14 |
| 高山柳 | *Salix cupularis* Rehd. | Salicaceae | 50 | 3.09 | 0.00 | 1.22 | 0.00 | 0.00 | 0.00 | 0.00 | 0.00 | 0.99 | 0.00 | 0.86 | 0.20 |
| 马尾松 | *Pinus massoniana* Lamb. | Pinaceae | 44 | 1.05 | 1.64 | 1.82 | 0.00 | 0.00 | 1.86 | 0.99 | 0.99 | 0.00 | 0.00 | 0.90 | 0.77 |
| 华北落叶松 | *Larix principis-rupprechtii* Mayr. | Pinaceae | 42 | 1.00 | 0.66 | 0.00 | 0.00 | 0.00 | 1.54 | 2.66 | 0.76 | 0.00 | 0.00 | 0.33 | 0.99 |
| 野核桃 | *Juglans cathayensis* Dode | Juglandaceae | 37 | 0.00 | 0.00 | 0.00 | 0.41 | 0.00 | 0.83 | 0.46 | 1.58 | 1.32 | 2.35 | 0.08 | 1.31 |
| 椴树 | *Tilia tuan* Szyszyl. | Tiliaceae | 31 | 1.50 | 0.74 | 0.26 | 0.00 | 0.00 | 2.50 | 0.00 | 0.93 | 0.97 | 0.00 | 0.50 | 0.88 |
| 山杨 | *Populus davidiana* Dode | Salicaceae | 30 | 0.00 | 0.00 | 0.27 | 4.20 | 0.00 | 0.53 | 0.00 | 0.00 | 0.00 | 0.00 | 0.89 | 0.11 |
| 湖北花楸 | *Sorbus hupehensis* Schneid. | Rosaceae | 29 | 0.68 | 0.00 | 0.00 | 0.00 | 2.03 | 0.00 | 0.00 | 0.00 | 0.00 | 0.00 | 0.54 | 0.00 |
| 梾木 | *Swida macrophylla* (Wall.) Sojak | Cornaceae | 29 | 0.68 | 0.20 | 0.26 | 0.27 | 0.27 | 0.00 | 0.77 | 0.50 | 0.77 | 0.71 | 0.34 | 0.55 |
| 青杄 | *Picea wilsonii* Mast. | Pinaceae | 26 | 1.04 | 2.66 | 0.00 | 0.00 | 1.02 | 0.43 | 0.00 | 0.00 | 0.00 | 0.00 | 0.94 | 0.09 |
| 千金榆 | *Carpinus cordata* Bl. | Betulaceae | 22 | 0.00 | 0.00 | 1.02 | 0.00 | 0.00 | 0.00 | 0.27 | 1.06 | 1.30 | 0.31 | 0.20 | 0.59 |
| 四照花 | *Dendrobenthamia japonica* (DC.) Fang  var. *chinensis* (Osborn) Fang | Cornaceae | 20 | 0.00 | 0.00 | 0.00 | 0.00 | 0.00 | 0.00 | 0.00 | 0.47 | 0.36 | 1.36 | 0.00 | 0.44 |
| 鸡爪槭 | *Acer palmatum* Thunb. | Aceraceae | 17 | 0.60 | 1.01 | 0.00 | 0.00 | 0.19 | 0.00 | 0.00 | 0.00 | 0.00 | 0.62 | 0.36 | 0.12 |
| 坚桦 | *Betula chinensis* Maxim. | Betulaceae | 15 | 0.00 | 0.88 | 0.39 | 0.00 | 0.53 | 0.94 | 0.00 | 0.00 | 0.00 | 0.00 | 0.36 | 0.19 |
| 冬瓜杨 | *Populus purdomii* Rehd. | Salicaceae | 12 | 0.00 | 0.00 | 0.00 | 1.91 | 0.00 | 0.00 | 0.00 | 0.00 | 0.00 | 0.00 | 0.38 | 0.00 |
| 刺叶高山栎 | *Quercus spinosa* David ex Franchet | Fagaceae | 11 | 0.00 | 0.00 | 0.00 | 0.16 | 0.18 | 0.00 | 0.00 | 0.73 | 0.23 | 0.00 | 0.07 | 0.19 |
| 灯台树 | *Bothrocaryum controversum* (Hemsl.) Pojark. | Cornaceae | 9 | 0.00 | 0.00 | 0.24 | 0.00 | 0.00 | 0.00 | 0.24 | 0.00 | 0.80 | 0.28 | 0.05 | 0.26 |
| 微毛樱桃 | *Cerasus clarofolia* (Schneid.) Yü et Li | Rosaceae | 8 | 0.00 | 0.00 | 0.00 | 0.00 | 0.00 | 0.00 | 0.40 | 0.00 | 0.00 | 0.00 | 0.00 | 0.08 |
| 山茱萸 | *Cornus officinalis* Sieb. et Zucc. | Cornaceae | 8 | 0.34 | 0.00 | 0.00 | 0.19 | 0.00 | 0.28 | 0.00 | 0.00 | 0.34 | 0.29 | 0.11 | 0.18 |
| 化香 | *Platycarya strobilacea* Sieb. et Zucc. | Juglandaceae | 7 | 0.00 | 0.00 | 0.00 | 0.00 | 0.00 | 0.00 | 0.00 | 0.46 | 0.00 | 0.43 | 0.00 | 0.18 |
| 八角枫 | *Alangium chinense* (Lour.) Harms | Alangiaceae | 6 | 0.29 | 0.32 | 0.11 | 0.00 | 0.00 | 0.00 | 0.00 | 0.00 | 0.00 | 0.00 | 0.14 | 0.00 |
| 华榛 | *Corylus chinensis* Franch. | Betulaceae | 4 | 0.00 | 0.00 | 0.00 | 0.00 | 0.00 | 0.00 | 0.25 | 0.00 | 0.00 | 0.00 | 0.00 | 0.05 |
| 泡花树 | *Meliosma cuneifolia* Franch. | Sabiaceae | 4 | 0.13 | 0.00 | 0.00 | 0.00 | 0.00 | 0.00 | 0.15 | 0.29 | 0.00 | 0.00 | 0.03 | 0.09 |
| 盐肤木 | *Rhus chinensis* Mill. | Anacardiaceae | 4 | 0.00 | 0.00 | 0.00 | 0.00 | 0.00 | 0.00 | 0.00 | 0.00 | 0.00 | 0.27 | 0.00 | 0.05 |
| 青蛙皮槭 | *Acer grosseri* pax | Aceraceae | 3 | 0.00 | 0.00 | 0.00 | 0.00 | 0.00 | 0.00 | 0.22 | 0.00 | 0.15 | 0.00 | 0.00 | 0.07 |
| 青麸杨 | *Rhus potaninii* Maxim. | Anacardiaceae | 2 | 0.00 | 0.00 | 0.00 | 0.00 | 0.00 | 0.00 | 0.00 | 0.00 | 0.00 | 0.27 | 0.00 | 0.05 |
| 陕甘黄毛槭 | *Acer fulvescens* Rehd. subsp. fupingense  (Fang et W.K.Hu) Fang et W.K.Hu | Aceraceae | 1 | 0.00 | 0.00 | 0.09 | 0.00 | 0.00 | 0.00 | 0.00 | 0.00 | 0.00 | 0.00 | 0.02 | 0.00 |
| 红豆杉 | *Taxus chinensis* (Pilger) Rehd. | Taxaceae | 1 | 0.00 | 0.00 | 0.00 | 0.00 | 0.11 | 0.00 | 0.00 | 0.00 | 0.00 | 0.00 | 0.02 | 0.00 |
